# Pandemic-Related Reductions in Respiratory Infections and New-Onset Nephrotic Syndrome: A Multicenter Cohort Study in Japan

**DOI:** 10.1016/j.xkme.2026.101452

**Published:** 2026-06-23

**Authors:** Kiyoshi Asakawa, Ken Iseri, Kosuke Nakano, Shinji Kobayashi, Taigi Yamazaki, Iori Taki, Noriko Hida

**Affiliations:** 1 Division of Clinical Research and Development, Department of Clinical Research and Development, Graduate School of Pharmacy, SHOWA Medical University, Tokyo, Japan; 2 Jinsei-kai Kasai Dialysis Clinic, Tokyo, Japan; 3 Center for Clinical Research and Development, National Center for Child Health and Development, Tokyo, Japan; 4 Nature Insight Co, Ltd, Tokyo, Japan

**Keywords:** Acetaminophen, COVID-19, infection, nephrotic syndrome

## Abstract

**Rationale & Objective:**

Infectious diseases have been associated with pediatric idiopathic nephrotic syndrome (NS). The effect of pandemic-related changes in infection exposure on NS epidemiology remains unclear.

**Study Design:**

Retrospective cohort study.

**Setting & Participants:**

Data from children aged 6 months to 15 years from 11 hospitals in Japan, extracted from the Pediatric Medical Information Collection System. Two periods were compared: prepandemic (April 2019-March 2020) and first pandemic year (April 2020-March 2021).

**Analytical Approach:**

Event counts (new-onset NS, relapse, respiratory tract infections, acetaminophen prescriptions, coronavirus disease 2019 diagnoses) were modeled using a negative binomial generalized linear mixed model with hospital-specific random intercepts. Sensitivity analyses used zero-inflated Poisson, Poisson mixed models, and quasi-Poisson.

**Results:**

There were 218 new-onset NS cases (127 prepandemic, 91 during the pandemic). The pandemic period was associated with a decrease in incidence, and estimates were similar across distributional sensitivity analyses, whereas extended time-window analyses attenuated statistical significance. The relapse rate among 366 patients in remission was 0.094 per person-year before the pandemic and 0.056 per person-year during the pandemic; the period effect was not statistically significant. Respiratory tract infections decreased by approximately half, and acetaminophen prescriptions declined. The number of coronavirus disease 2019 diagnoses increased sharply.

**Limitations:**

Observational design limits causal inference. Findings may not generalize beyond Japanese populations.

**Conclusions:**

Reduced respiratory infection exposure during the pandemic may be associated with lower new-onset NS incidence. Preventive measures against respiratory infections may help reduce NS occurrence.

## Plain-Language Summary

Idiopathic nephrotic syndrome is a childhood kidney disease that causes edema and protein loss in the urine. Some relapses occur after common colds. During the coronavirus disease 2019 pandemic, masking and distancing reduced many respiratory infections, offering a chance to study how infection exposure relates to this disease. We used electronic medical records from pediatric hospitals in Japan to compare new diagnoses and relapses in the year before the pandemic with those in the first pandemic year, and we also tracked respiratory infection visits and use of fever medicine. New diagnoses were lower during the pandemic year, whereas relapses showed a smaller, uncertain change. Preventing respiratory infections may help reduce some cases and improve care for vulnerable children.

Idiopathic nephrotic syndrome (hereafter referred to as NS), characterized by hypoalbuminemia resulting from excessive proteinuria, is one of the most common glomerulopathies in childhood.^1^, ^2^, ^3^ NS often presents with edema, hypertension, and sometimes acute kidney injury, frequently necessitating steroid therapy. Although most children respond to corticosteroids, the clinical course is heterogeneous, with recurrent relapses in a substantial proportion and a meaningful risk of complications such as infection and thrombosis. Its pathogenesis is not fully understood, although immune dysregulation may play a key role.^4^ Clinical studies have shown that upper respiratory tract infections (common colds), school events, and psychological stress are triggers for relapse in childhood NS, and similar exposures are hypothesized to contribute to disease onset.^5^ Considering the above, population-level fluctuations in infectious exposures may influence the incidence and relapse dynamics of NS.

The coronavirus disease 2019 (COVID-19) pandemic, caused by coronavirus 2 SARS-CoV-2) infection, brought unprecedented changes in children’s exposures, including lockdowns, school closures, and physical distancing to curb viral transmission. These measures, although targeted at SARS-CoV-2, broadly suppressed other respiratory viruses.^6^, ^7^, ^8^ In Belgium and the United Kingdom, respiratory syncytial virus infections decreased by >98%, pneumococcal infections by 68%-82%, and influenza cases by >90%.^6^^,^^9^ Similarly, Denmark reported a 36% reduction in infectious disease-related hospitalizations among children during the initial lockdown period compared to 2018-2019.^10^ Unlike many Western countries, Japan did not impose legally enforced lockdowns but implemented nonpharmacologic interventions (NPIs) such as masking, school closures, and physical distancing. These actions may reduce the incidence of infectious diseases, but they may also introduce psychological stress. To our knowledge, only a few studies have investigated changes in the incidence of NS during the pandemic and no study from Asia.^11^^,^^12^ Moreover, the Japanese context—marked by high adherence to NPIs^13^^,^^14^ and early nationwide emergency declarations—may offer a distinctive setting to investigate pandemic-related effects on pediatric glomerulopathies at scale. Understanding these effects is clinically important: if reductions in community infections lower the risks of onset and relapses, preventive strategies that limit infectious exposures could yield benefits extending beyond the pandemic.

Thus, the aim of the present study was to investigate changes in the incidence of new-onset NS before and after the COVID-19 emergency declarations using a nationwide database in Japan. Furthermore, we explored differences in NS relapses among children in remission before the pandemic, as well as contemporaneous changes in respiratory tract infections, acetaminophen prescribing (as a pragmatic indicator of febrile respiratory illness and symptomatic management), and COVID-19 incidence, to clarify whether infection prevention might suppress NS onset and relapse at the population level.

## Methods

### Study Population and Design

Data were obtained from the Pediatric Medical Information Collection System (P-MICS), established through the Pediatric Drugs Information Collection Network Development Project, subsidized by the Ministry of Health, Labour, and Welfare. P-MICS collects electronic medical records from 11 participating hospitals and clinics nationwide to investigate pediatric practices including actual use of medicines in children and the evaluation of their safety. The database contains prescription data, laboratory results, hospitalization records, and clinical diagnoses of more than 1,130,000 children in Japan, coded according to the *International Classification of Diseases, Tenth Revision* (ICD-10). The study protocol was approved by the Showa University Ethics Committee (approval number: 2023-309-A). The need for informed consent was waived because the information was deidentified.

We conducted a retrospective cohort study using data from 11 hospitals between April 2019 and March 2021. Based on Japan’s emergency declaration timeline (initial declaration on April 7, 2020, for 7 prefectures, extended nationwide on April 16), we defined the prepandemic period as April 2019 to March 2020 and the pandemic period as April 2020 to March 2021. Participants were children aged 6 months to 15 years. These April to March windows were chosen to capture complete annual cycles and thereby minimize potential confounding by seasonality. For sensitivity analyses evaluating the robustness of results to the selected comparison windows, we additionally extracted data from October 2018 through September 2021 from the same 11 hospitals.

### Study Outcomes

The primary outcome was new-onset NS. Secondary outcomes included NS relapses, respiratory tract infections, acetaminophen prescriptions, and COVID-19 diagnosis. KDIGO (Kidney Disease: Improving Global Outcomes) guidance emphasizes standardized assessment of proteinuria in children, accounting for age and body size.^15^ However, in this multicenter electronic medical records database, proteinuria results were recorded heterogeneously across institutions, and body-surface-area–indexed values were not available. Therefore, we adopted a pragmatic operational definition combining ICD-10 diagnosis codes with laboratory evidence of hypoalbuminemia and nephrotic-range proteinuria, applied consistently across centers and study periods.

New-onset NS was defined as patients without prior NS diagnosis before March 31, 2019, who subsequently developed ICD-10 code N04 during the observation period, accompanied by serum albumin ≤3.0 g/dL and proteinuria (≥2.0 g/g creatinine [cr] or qualitative ≥3+ on single measurement).^16^ For time-window sensitivity analyses with an earlier start date, the ‘no prior NS diagnosis’ criterion was anchored to the start of each corresponding window, while the ICD-10 and laboratory criteria were kept identical across analyses.

**Figure 1:**
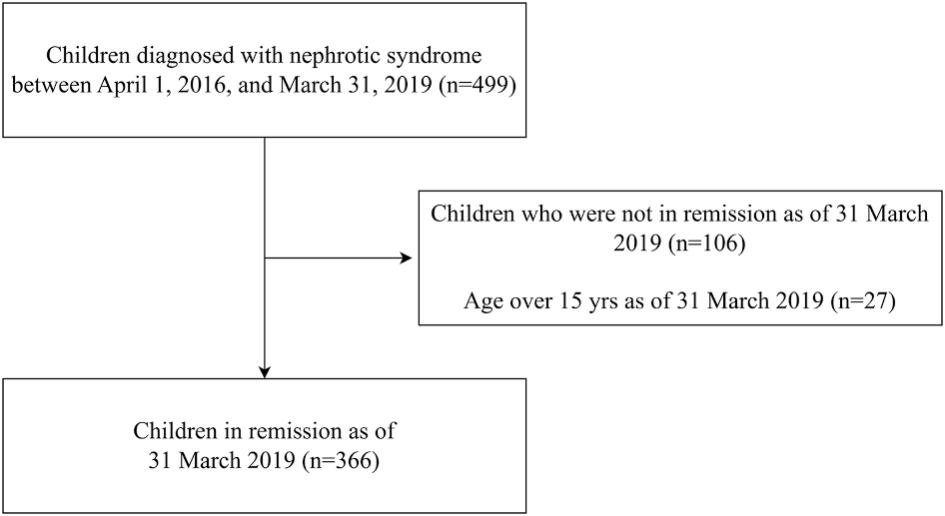
Patient disposition for the relapse outcome. Among 499 children diagnosed with nephrotic syndrome between April 1, 2016 and March 31, 2019, age and remission status were assessed as of March 31, 2019 (data cutoff). Twenty-seven children were aged older than 15 years at the cutoff and were excluded from the pediatric cohort. Of the remaining 472, 106 (22.5%) were not in remission and 366 (77.5%) were in remission.

For NS relapse analysis, we identified 499 children diagnosed with NS (ICD-10 code N04, serum albumin ≤3.0 g/dL, and proteinuria ≥2.0 g/d or qualitative ≥3+) between April 1, 2016 and March 31, 2019. Of these, 366 children in remission as of March 31, 2019 were included (Fig 1). Remission was defined as proteinuria <0.2 g/g cr or negative qualitative test on ≥3 occasions.^16^ In this study, we considered remission as a proxy for complete remission; partial remission could not be uniformly ascertained because the timing of urine sampling (first-morning vs random) was not available.

Relapse during the observation period was defined as serum albumin ≤3.0 g/dL and proteinuria (≥2.0 g/g cr or qualitative ≥3+). Respiratory tract infections and COVID-19 were identified using corresponding ICD-10 codes during the observation period (Table S1). Acetaminophen prescriptions were identified by searching for the term ‘acetaminophen.’

### Statistical Analysis

For new-onset NS, respiratory tract infections, acetaminophen prescriptions, and COVID-19 diagnoses, we modeled event counts with generalized linear mixed models (GLMMs) using a negative binomial distribution to accommodate overdispersion. Each model included period (prepandemic vs pandemic) as a fixed effect and a hospital-level random intercept:

Events∼Period+(1∣Hospital).

Incidence rate ratios (IRRs) and 95% confidence intervals (CIs) were obtained by exponentiating model coefficients.

To assess the robustness of our findings, we performed prespecified sensitivity analyses: (1) zero-inflated Poisson models to address excess zeros in monthly or hospital-level series; (2) Poisson mixed models with the same random-effects structure; and (3) quasi-Poisson regression for variance misspecification. In addition to model-based sensitivity analyses, we conducted time-window sensitivity analyses to evaluate potential sensitivity to the choice of comparison windows (including seasonality and secular changes). Keeping April 2020 as the interruption point, we repeated the primary negative binomial GLMM using the following: (1) an extended prepandemic period (October 2018-March 2020) compared with the same pandemic period (April 2020-March 2021), and (2) 2 equal-length 18-month periods (October 2018-March 2020 vs April 2020-September 2021).

For the relapse analysis, outcomes were counts of relapse episodes per patient within each period, with person-time computed after excluding days spent in relapse. The primary relapse model was a negative binomial GLMM with a hospital-level random intercept and a log(person-years) offset.

$${\text{Relapse}\;\text{events}}_{i}∼{\text{Period}}_{i}+\left(1|{\text{Hospital}}_{i}\right)+\text{offset}\left\{\log\left({\text{PY}}_{i}\right)\right\}\text{.}$$

Sensitivity analyses for relapse included the following: (1) a negative binomial GLMM with both patient- and hospital-level random intercepts to account for within-patient clustering; and (2) a patient fixed-effects Poisson model restricted to patients with at least 1 relapse, with a log(PY) offset and standard errors clustered at the patient level. As an exploratory subgroup analysis, we repeated the primary relapse model stratified by age (<6 vs ≥6 years).

Statistical analyses were performed using R for Windows, version 4.4.2 (The R Foundation for Statistical Computing). Statistical significance was set at *P* < 0.05.

## Results

### New-Onset NS

**Figure 2:**
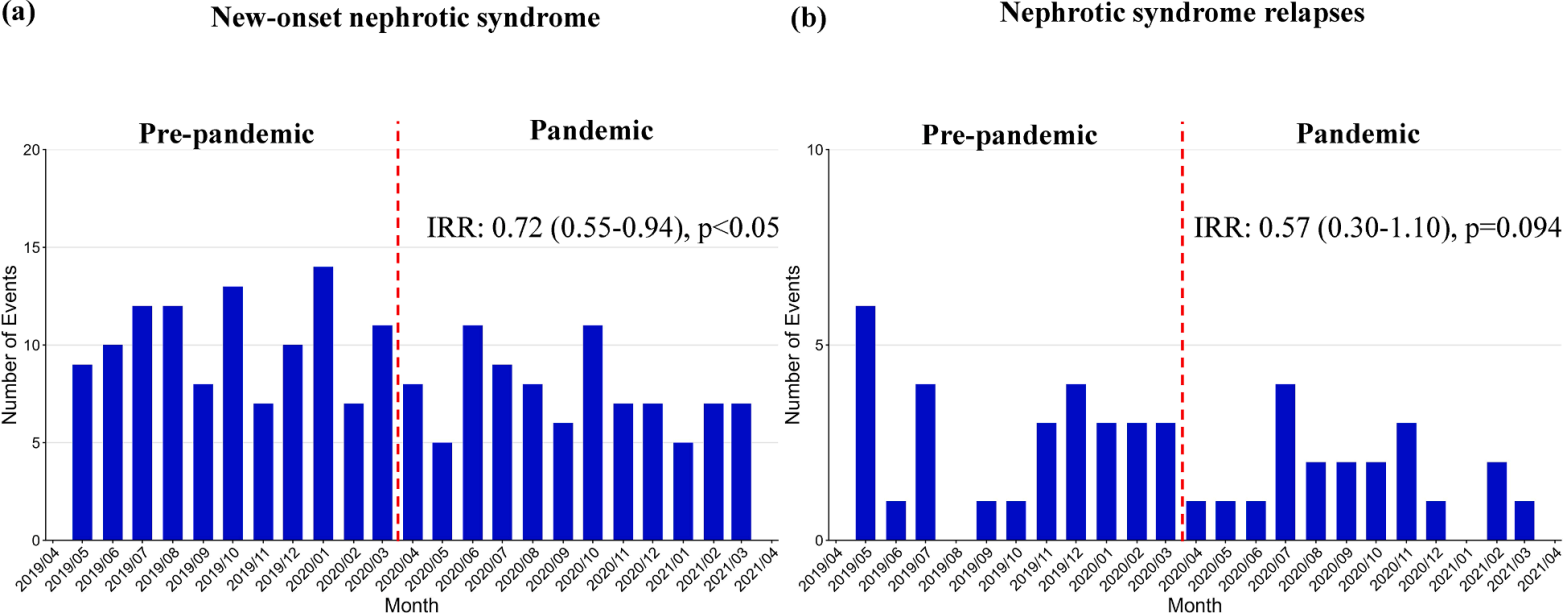
Incidence and relapse of pediatric idiopathic nephrotic syndrome before versus during the coronavirus disease 2019 (COVID-19) pandemic in Japan. (A) New-onset nephrotic syndrome. (B) Nephrotic syndrome relapses among children in remission before the observation window. Bars display counts of interest across 11 participating hospitals in prepandemic (April 2019-March 2020) and pandemic (April 2020-March 2021) periods. Text shows incidence rate ratios (IRRs) with 95% confidence intervals and two-sided *P* values from a generalized linear mixed model.

There were 218 new cases of NS during the 2 observation periods: 127 prepandemic (median age 5 years, interquartile range, 2-10 years) and 91 during the pandemic (median age 5 years, interquartile range, 3-8 years) (Fig 2A). In the primary negative binomial GLMM, the pandemic period was associated with a lower incidence of new-onset NS (IRR, 0.72; 95% CI, 0.55-0.94; *P* < 0.05). Estimates were similar in distributional sensitivity analyses: zero-inflated Poisson (IRR, 0.72; 95% CI, 0.54-0.94; *P* < 0.05), Poisson mixed model (IRR, 0.72; 95% CI, 0.55-0.94; *P* < 0.05), and quasi-Poisson (IRR, 0.72; 95% CI, 0.56-0.92; *P* < 0.01). In time-window sensitivity analyses, the point estimates remained <1.0, but CIs crossed unity; extending the prepandemic period to 18 months (October 2018-March 2020 vs April 2020-March 2021) yielded an IRR of 0.795 (95% CI, 0.622-1.017; *P* = 0.07), and extending both periods to 18 months (October 2018- March 2020 vs April 2020-September 2021) yielded an IRR of 0.836 (95% CI, 0.675-1.036; *P* = 0.10).

### Relapse of HS

Among 366 children (median age 7 years, interquartile range, 5-10 years) who were in remission as of March 31, 2019, we found 34 relapses during the prepandemic period and 20 during the pandemic (person-years: 360.4 and 355.2, respectively), corresponding to relapse rates of 0.094 and 0.056 per person-year. In the primary model, the pandemic period was not significantly associated with relapse risk (IRR, 0.57; 95% CI, 0.30-1.10; *P* = 0.09) (Fig 2B). Sensitivity analyses showed similar results: GLMMs with both patient- and hospital-level random intercepts (IRR, 0.63; 95% CI, 0.35-1.10; *P* = 0.11) and Poisson model (IRR, 0.69; 95% CI, 0.35-1.35; *P* = 0.28). In age-stratified analyses (<6 vs ≥6 years), the pandemic period was associated with fewer events in both age groups, although these findings did not reach statistical significance (<6 years: IRR, 0.605; 95% CI, 0.207-1.772; ≥6 years: IRR, 0.556; 95% CI, 0.246-1.253).

### Respiratory Tract Infections

**Figure 3:**
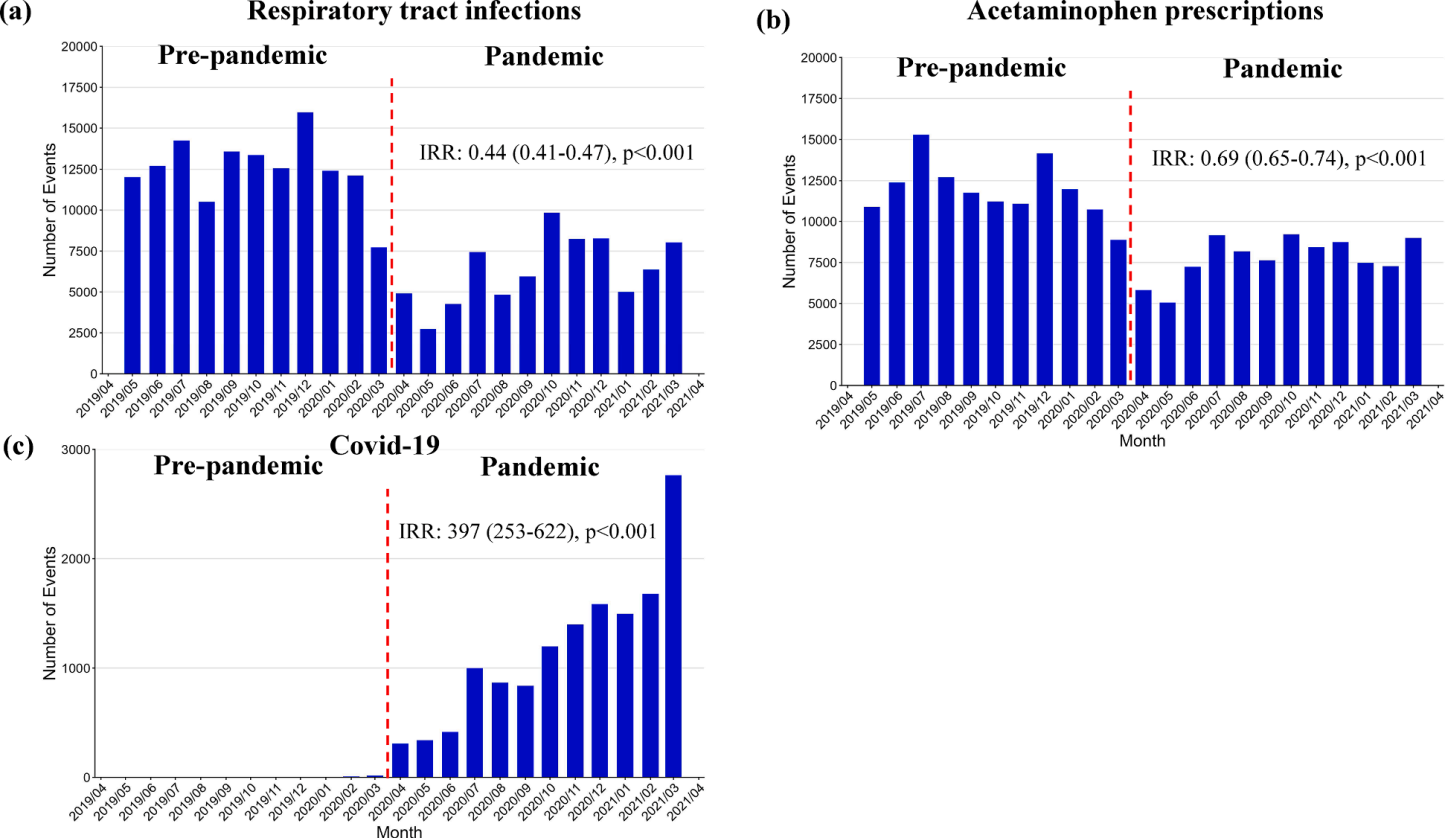
Changes in respiratory tract infections, acetaminophen prescriptions, and COVID-19 before versus during the COVID-19 pandemic in Japan. (A) Respiratory tract infections. (B) Acetaminophen prescriptions. (C) COVID-19 diagnoses. Bars display counts of interest across 11 participating hospitals in prepandemic (April 2019-March 2020) and pandemic (April 2020-March 2021) periods. Text shows IRRs with 95% confidence intervals and two-sided *P* values from a generalized linear mixed model. Abbreviations: COVID-19, coronavirus disease 2019; IRR, incidence rate ratio.

Monthly totals of respiratory tract infections decreased from 149,534 prepandemic to 75,876 during the pandemic (mean per month, 12,461 to 6,323; ratio 0.51) (Fig 3A). Thus, respiratory infections approximately halved during the pandemic year. The robustness of this result was confirmed by sensitivity analyses: zero-inflated Poisson (IRR, 0.51; 95% CI, 0.50-0.51; *P* < 0.001), Poisson mixed model (IRR, 0.51; 95% CI, 0.50-0.51; *P* < 0.001), and quasi-Poisson (IRR, 0.51; 95% CI, 0.47-0.54; *P* < 0.001).

### Acetaminophen Prescriptions

There were 142,331 prescriptions prepandemic and 93,200 during the pandemic. The pandemic period was associated with a pronounced reduction (IRR, 0.69; 95% CI, 0.65-0.74; *P* < 0.001) (Fig 3B). Sensitivity analyses showed similar results: zero-inflated Poisson (IRR, 0.65; 95% CI, 0.65-0.66; *P* < 0.001), Poisson mixed model (IRR, 0.65; 95% CI, 0.65-0.66; *P* < 0.001), and quasi-Poisson (IRR, 0.65; 95% CI, 0.62-0.69; *P* < 0.001).

### COVID-19

Thirty COVID-19 diagnoses were recorded near the end of the prepandemic period; during the pandemic year, there were 13,893 diagnoses. Fig 3C shows a marked increase in incidence after Japan’s emergency declaration. GLMM analysis showed that the pandemic period was strongly associated with higher incidence (IRR, 396.9; 95% CI, 253.2-622.1; *P* < 0.001), with comparable magnitudes in sensitivity analyses: zero-inflated Poisson (IRR, 421; 95% CI, 286-621; *P* < 0.001), Poisson mixed model (IRR, 463; 95% CI, 324-663; *P* < 0.001), and quasi-Poisson (IRR, 463; 95% CI, 74-2,894; *P* < 0.001).

## Discussion

In this study of a multicenter cohort from 11 hospitals in Japan, the incidence of new-onset NS in children declined during the COVID-19 pandemic year compared with the prior year (IRR, 0.72; 95% CI, 0.55-0.94). In time-window sensitivity analyses to address potential seasonality and secular changes, the point estimates remained <1.0 but did not reach statistical significance when longer windows were used. Among children in remission before the pandemic, relapse rates of NS in pandemic period appeared to be lower than that in prepandemic period without reaching statistical significance (IRR, 0.57; 95% CI, 0.30-1.10). Concurrently, respiratory tract infections were reduced by approximately 50%, and acetaminophen prescriptions declined markedly, suggesting a broad reduction in community respiratory illness burden. These findings indicated a population-level association between infectious exposures and disease activity in pediatric NS.

Our findings regarding incident NS align with recent European data. A study from the Netherlands and Paris region found that although overall annual incidence of first-onset idiopathic NS did not differ substantially between prepandemic and pandemic years, incidence decreased significantly during school closure periods (0.53 vs 1.31 per 100,000 in the Netherlands; 0.94 vs 2.63 per 100,000 in Paris).^11^ Conversely, a European multicenter cohort found no significant change in relapse rates during 2020 versus the prior 5 years and reported similar numbers of new NS cases during 2018-2020.^17^ Additionally, a US single-center cohort reported lower relapse rates during social distancing compared with the prepandemic baseline.^12^ On the other hand, a population-based study from Fukushima (2006-2016) showed an average annual pediatric NS incidence of approximately 5.16 per 100,000 (range, 3.47-9.26), and incidence peaked in 2011, which was the year of the Great East Japan Earthquake and Fukushima nuclear power plant accident. They concluded that infections and steroid withdrawal were among the most common relapse triggers.^18^ Crowded communal sheltering necessitated by the earthquake likely facilitated transmission of infectious diseases, in contrast with the physical-distancing measures widely implemented during the COVID-19 pandemic.

Our findings in NS parallel similar patterns observed across pediatric respiratory infections during the pandemic. Studies from the Italy and Denmark reported significant reductions in community-acquired respiratory infections and infection-related pediatric hospitalizations during lockdown, consistent with the diminution of seasonal epidemics during school closures.^10^^,^^19^ Multinational surveillance showed substantial reductions in early 2020 in invasive diseases caused by *Streptococcus pneumoniae*, *Haemophilus influenzae*, and *Neisseria meningitidis* coinciding with containment measures (eg, pneumococcal disease IRR ≈0.32 at 4 weeks and 0.18 at 8 weeks after movement reductions).^20^ In our study, during the pandemic, although COVID-19 case numbers increased, both the number of upper respiratory tract infection diagnoses and acetaminophen prescriptions decreased during the pandemic period, indicating a general decline in infectious diseases overall from a broader perspective. These independent infection data during the pandemic suggest that infection control measures play a key role in reducing both respiratory infections in the community. Our findings, showing similar declining trends in respiratory infections and incident NS when social mixing was most restricted, support a mechanistic association between reduced respiratory virus transmission and lower NS incidence. A chronological prospective study showed that upper respiratory tract infections, particularly the ‘common cold,’ were the predominant triggers for relapse in children with steroid-dependent and frequently relapsing NS, followed by school-related events.^5^ A review analyzing studies conducted in Pakistan, Canada, Japan, and India similarly indicated that at least half of NS relapses occur after viral upper respiratory tract infections.^4^ Although the pathogenesis of NS triggered by upper respiratory tract infection has not been fully elucidated, increased circulating pulmonary surfactant, activation of podocyte Toll-like receptor pathways, and immune dysregulation—characterized by reduced regulatory T cells and expansion of Th17 cells—may contribute to its pathogenesis.^1^^,^^21^, ^22^, ^23^ As for changes in health care-seeking behavior during the pandemic, a study from a population-based database showed a substantial reduction in pediatric health care utilization (24% for first outpatient visits, 7% for follow-up visits, and 2.6% for patient-days of hospitalizations) in Japan.^24^ However, the magnitude of the reduction in hospitalizations for specific infectious diseases was far greater than what could be explained by health care avoidance alone. In a remote prefecture in Japan, where the incidence of COVID-19 in pediatric patients was extremely low and access to alternative medical facilities was limited, a marked reduction in hospitalizations for infectious diseases such as influenza (−74.8%) and respiratory syncytial virus infection (−93.5%) was reported.^25^ The limited availability of alternative health care facilities in this setting makes it unlikely that the observed reduction was due to patients shifting to other institutions. These findings may suggest that the observed decrease in pediatric respiratory infections during the COVID-19 pandemic was largely because of an actual reduction brought about by NPIs, rather than an apparent decrease stemming from changes in health care-seeking behavior or shifts in health care utilization patterns. Furthermore, in cases of NS presenting with sudden lower limb edema, it is unlikely that patients would entirely refrain from seeking medical attention. Indeed, although a small study showed a somewhat longer time between noticing symptoms of NS and diagnosis after the COVID-19 pandemic (7 days vs 3.5 days), this modest delay suggests that although health care-seeking behavior may have been partially affected, the effect was limited and unlikely to fully account for changes in NS incidence.^26^ Taken together with accumulating evidence, our results suggest that NPIs may reduce both incidence and recurrence rates.

The relapse estimate (IRR, 0.57) was directionally consistent with incident NS (IRR, 0.72), with overlapping CIs, indicating limited precision (fewer events) rather than a clear dissociation. Relapse risk is also influenced by treatment factors (eg, steroid taper/withdrawal) and may be buffered by maintenance immunosuppression and short courses of daily prednisolone during upper respiratory tract infections. In an Omicron-era cohort, SARS-CoV-2 infection became common in children with NS, but clinically relevant relapses during infection were uncommon.^27^ Our database comprises core pediatric hospitals within each region. However, the limited sample size may result in an underpowered analysis. Another possible explanation for this discrepancy is that children in remission (relapse study cohort) may already have been receiving immunosuppressive medications, in contrast to newly diagnosed children, and these medications may have influenced these differences. In addition, clinical practices may have attenuated relapse risk even when respiratory infection exposure occurred. International guidelines recommending short periods of daily prednisolone during upper respiratory tract infections have been associated with lower infection-related relapses, potentially buffering the effect of residual respiratory infections.^28^^,^^29^

Strengths of our study include the use of one of the largest pediatric databases in Japan. Moreover, defining NS cases using ICD-10 codes, blood tests, and urine tests was likely to be highly reliable.

However, some limitations should be discussed when interpreting the results of the present study. First, as in any observational study, we cannot conclude on causality. In particular, the role of common respiratory infections as a trigger for new-onset NS is not an established paradigm and remains hypothetical. SARS-CoV-2 infection also could be a potential immunologic trigger for new-onset NS and/or relapse similar to common respiratory infections.^30^ Early serology in immunosuppressed pediatric kidney cohorts from Italy showed low SARS-CoV-2 IgG positivity, suggesting limited exposure early in the pandemic.^31^ Although COVID-19 cases surged during the pandemic in Japan, the number of patients with COVID-19 remained low when viewed within the broader context of respiratory infectious diseases. Overall, respiratory infectious diseases appear to have declined during the pandemic period. Second, case ascertainment relied on encounters within the P-MICS network; care received outside the network and asymptomatic SARS-CoV-2 infections were not captured. We did not collect center-level information on changes in admission thresholds or diagnostic workflows during the pandemic, which could have influenced ascertainment. Third, generalizability may be limited because Japan’s sociocultural context and the implementation/adherence to NPIs differs from those in other countries. Pandemic-related changes in pediatric health care utilization may differ across health care systems, potentially modifying case ascertainment. In addition, our database did not capture the timing of urine collection (eg, first-morning vs random spot urine). Therefore, we could not apply guideline-based definitions to distinguish complete from partial remission, which may have led to misclassification of remission status. Also, we could not perform stratified analyses for new-onset NS because the number of events was limited, and subgrouping would have led to unstable estimates. Fourth, our inference for new-onset NS may be sensitive to the definition of the comparison windows. Although the April to March windows were selected a priori to align with Japan’s first state of emergency and to capture complete annual cycles, extending the baseline and/or follow-up attenuated statistical significance, underscoring limited statistical power and potential year-to-year variability. Future studies with larger populations and analytical approaches that explicitly model temporal trends are required.

In conclusion, a lower incidence of new-onset pediatric NS was observed during the first pandemic year compared with the preceding year, alongside marked reductions in respiratory tract infection diagnoses and acetaminophen prescribing. However, time-window sensitivity analyses attenuated statistical significance, indicating uncertainty in the magnitude of change. Taken together, these findings support a population-level association between infectious exposures and disease activity in pediatric NS and suggest that NPIs may have contributed to reduced NS incidence through suppression of common respiratory infections.

## References

[1] Vivarelli M., Massella L., Ruggiero B., Emma F. (2017). Minimal change disease. Clin J Am Soc Nephrol.

[2] Noone D.G., Iijima K., Parekh R. (2018). Idiopathic nephrotic syndrome in children. Lancet.

[3] Chan E.Y.H., Boyer O. (2025). Childhood idiopathic nephrotic syndrome: recent advancements shaping future guidelines. Pediatr Nephrol.

[4] Uwaezuoke S.N. (2015). Steroid-sensitive nephrotic syndrome in children: triggers of relapse and evolving hypotheses on pathogenesis. Ital J Pediatr.

[5] Takahashi S., Wada N., Murakami H. (2007). Triggers of relapse in steroid-dependent and frequently relapsing nephrotic syndrome. Pediatr Nephrol.

[6] Van Brusselen D., De Troeyer K., Ter Haar E. (2021). Bronchiolitis in COVID-19 times: a nearly absent disease?. Eur J Pediatr.

[7] Chen W., Wang H., Ten X. (2025). Impact of COVID-19 control measures on influenza positivity among patients with acute respiratory infections, 2018-2023: an interrupted time series analysis. BMC Infect Dis.

[8] Takashita E., Shimizu K., Kawakami C. (2025). Impact of COVID-19 on respiratory virus infections in children, Japan, 2018-2023. Immun Inflamm Dis.

[9] Nagakumar P., Chadwick C.L., Bush A., Gupta A. (2021). Collateral impact of COVID-19: why should children continue to suffer?. Eur J Pediatr.

[10] Polcwiartek L.B., Polcwiartek C., Andersen M.P. (2021). Consequences of coronavirus disease-2019 (COVID-19) lockdown on infection-related hospitalizations among the pediatric population in Denmark. Eur J Pediatr.

[11] Veltkamp F., Thenot V., Mussies C. (2023). Incidence of idiopathic nephrotic syndrome during the Covid-19 pandemic in the Paris area (France) and in the Netherlands. Pediatr Nephrol.

[12] Crane C., Bakhoum C., Ingulli E. (2022). Rates of idiopathic childhood nephrotic syndrome relapse are lower during the COVID-19 pandemic. Pediatr Nephrol.

[13] Kusama T., Takeuchi K., Tamada Y., Kiuchi S., Osaka K., Tabuchi T. (2023). Compliance trajectory and patterns of COVID-19 preventive measures, Japan, 2020-2022. Emerg Infect Dis.

[14] Anan T., Ishimaru T., Hino A. (2023). CORoNaWork project. Association between COVID-19 infection rates by region and implementation of non-pharmaceutical interventions: a cross-sectional study in Japan. J Public Health (Oxf).

[15] Floege J., Gibson K.L., Kidney Disease: Improving Global Outcomes (KDIGO) Nephrotic Syndrome In Children Work Group (2025). KDIGO 2025 clinical practice guideline for the management of nephrotic syndrome in children. Kidney Int.

[16] Trautmann A., Boyer O., Hodson E., International Pediatric Nephrology Association (2023). IPNA clinical practice recommendations for the diagnosis and management of children with steroid-sensitive nephrotic syndrome. Pediatr Nephrol.

[17] Chiodini B., Bellotti A.S., Morello W. (2023). Relapse rate in children with nephrotic syndrome during the SARS-CoV-2 pandemic. Pediatr Nephrol.

[18] Kume Y., Kawasaki Y., Suyama K. (2021). Incidence and relapse triggers of childhood idiopathic nephrotic syndrome between 2006 and 2016: a population-based study in Fukushima, Japan. Tohoku J Exp Med.

[19] Rotulo G.A., Percivale B., Molteni M. (2021). The impact of COVID-19 lockdown on infectious diseases epidemiology: the experience of a tertiary Italian Pediatric Emergency Department. Am J Emerg Med.

[20] Brueggemann A.B., Jansen van Rensburg M.J., Shaw D. (2021). Changes in the incidence of invasive disease due to *Streptococcus pneumoniae, Haemophilus influenzae*, and *Neisseria meningitidis* during the COVID-19 pandemic in 26 countries and territories in the Invasive Respiratory Infection Surveillance Initiative: a prospective analysis of surveillance data. Lancet Digit Health.

[21] Lai K.W., Wei C.L., Tan L.K. (2007). Overexpression of interleukin-13 induces minimal-change-like nephropathy in rats. J Am Soc Nephrol.

[22] Cara-Fuentes G., Andres-Hernando A., Bauer C. (2022). Pulmonary surfactants and the respiratory-renal connection in steroid-sensitive nephrotic syndrome of childhood. iScience.

[23] Purohit S., Piani F., Ordoñez F.A., de Lucas-Collantes C., Bauer C., Cara-Fuentes G. (2021). Molecular mechanisms of proteinuria in minimal change disease. Front Med (Lausanne).

[24] Ohnishi T., Kinoshita M., Narumi S. (2025). Impact of COVID-19 on paediatric care in Japan: analysis of national health insurance claims data. Acta Paediatr.

[25] Shichijo K., Takeuchi S., Tayama T. (2021). Patient attendance at a pediatric emergency referral hospital in an area with low COVID-19 incidence. PLoS One.

[26] Watanabe Y., Fuyama M., Abe Y., Watanabe T., Ikeda H. (2023). Delayed diagnosis and exacerbation of hyperlipidemia in idiopathic nephrotic syndrome in children during the COVID-19 pandemic. Clin Exp Nephrol.

[27] Morello W., Vianello F.A., Bulgaro C., Montini G. (2023). Epidemiology, severity, and risk of SARS-CoV-2-related relapse in children and young adults affected by idiopathic nephrotic syndrome: a retrospective observational cohort study. Pediatr Nephrol.

[28] Abeyagunawardena A.S., Thalgahagoda R.S., Dissanayake P.V. (2017). Short courses of daily prednisolone during upper respiratory tract infections reduce relapse frequency in childhood nephrotic syndrome. Pediatr Nephrol.

[29] Rovin B.H., Adler S.G., Barratt J. (2021). Executive summary of the KDIGO 2021 guideline for the management of glomerular diseases. Kidney Int.

[30] Morello W., Vianello F.A., Proverbio E., Peruzzi L., Pasini A., Montini G. (2022). COVID-19 and idiopathic nephrotic syndrome in children: systematic review of the literature and recommendations from a highly affected area. Pediatr Nephrol.

[31] Morello W., Mastrangelo A., Guzzo I., COVID-19 Task Force of the Italian Society of Pediatric Nephrology (2021). Prevalence of SARS-CoV-2-IgG antibodies in children with CKD or immunosuppression. Clin J Am Soc Nephrol.

