# Supplementary material for: Pandemic-Related Reductions in Respiratory Infections and New-Onset Nephrotic Syndrome: A Multicenter Cohort Study in Japan
Source: Kidney Med. 2026 Jun 23;8(9):101452. doi: 10.1016/j.xkme.2026.101452 (PMC13471244; doi:10.1016/j.xkme.2026.101452)
Supplement: Supplementary file 1 [file mmc1.pdf]

Supplementary Material

Table S1. Definition of respiratory tract infections and COVID-19 by International Classification of Disease 10th Edition (ICD-10) codes.

**Variable**	**ICD-10 codes**
Respiratory tract infections	J00-J06、J09-J18、J20-J22
COVID-19	B34.2、U07.1

Abbreviations: ICD-10, International Classification of Disease – 10th Edition
